# Physiological Benefits, Applications, and Future Directions of β-Hydroxy-β-Methylbutyrate (HMB) in Food and Health Industries

**DOI:** 10.3390/foods14081294

**Published:** 2025-04-08

**Authors:** Sijing Zhou, Guijun Liu, Zhong Wang, Ziteng Lei, Wei Chen, Chengtao Wang

**Affiliations:** 1 Key Laboratory of Geriatric Nutrition and Health, Beijing Advanced Innovation Center for Food Nutrition and Human Health, Beijing Engineering and Technology Research Center of Food Additives, Ministry of Education, Beijing Technology and Business University (BTBU), Beijing 100048, China; 2150021014@st.btbu.edu.cn (S.Z.); liu_guijun01@163.com (G.L.); 2431031063@st.btbu.edu.cn (Z.W.); 2431032108@st.btbu.edu.cn (Z.L.); 2 Beijing Academy of Science and Technology, Beijing 100089, China; 3 Natural History Museum of China, Beijing 100050, China

**Keywords:** β-hydroxy-β-methylbutyrate (HMB), muscle growth, bio-manufacturing, food industry, sarcopenia

## Abstract

β-Hydroxy-β-methylbutyrate (HMB), a metabolite of the essential amino acid leucine, is acknowledged for its powerful role in facilitating muscle protein synthesis, reducing muscle catabolism, and promoting fat-free mass accumulation. With well-documented anticatabolic, anabolic, and lipolytic effects, HMB has been extensively studied in clinical settings and has exhibited potential in mitigating muscle loss induced by aging, cancer cachexia, and sarcopenia. Moreover, HMB finds applications in specialized medical nutrition, sports nutrition, and animal husbandry, with recent research illustrating its benefits in enhancing animal growth and immunity. This review highlights the current understanding of HMB’s physiological mechanisms, its diverse applications, and recent advancements in detection methods such as High-Performance Liquid Chromatography (HPLC), Gas Chromatography (GC), and Liquid Chromatography–Mass Spectrometry (LC–MS). Additionally, it discusses the future prospects of HMB bio-manufacturing. The establishment of standardized guidelines for its safe use and testing is crucial for its broader adoption in the food industry. Future research should focus on further elucidating HMB’s muscle growth mechanisms and broadening its applications across the food, health, and agricultural sectors. In sum, future studies should prioritize mechanistic exploration, safety and synergy, along with standardization to fully harness HMB’s potential.

## 1. Introduction

Aging has emerged as a critical global challenge in the 21st century. According to the World Population Prospects 2023, the proportion of individuals aged 65 and older is projected to increase dramatically by 2050, rising from 20% to 28% in developed nations, 9% to 17% in developing countries, and 3.7% to 6.1% in the least developed regions [1]. As the aging trend intensifies, health-related issues among the elderly are drawing increased attention. Epidemiological studies across diverse populations indicate that sarcopenia—the age-related loss of muscle mass and strength—affects 5.5% to 25.7% of adults aged 60 years or older. This condition has been identified as a major contributor to injury-related mortality among individuals aged 65 and above, profoundly impairing physical independence and quality of life. Furthermore, sarcopenia is strongly associated with elevated risks of all-cause mortality, cardiopulmonary comorbidities, neurocognitive decline, and adverse clinical outcomes, including falls, disability, and functional decline [2,3].

In response to these challenges, β-hydroxy-β-methylbutyrate (HMB) has emerged as a promising therapeutic candidate due to its well-documented muscle-preserving effects [4,5]. HMB, a five-carbon organic acid, is synthesized endogenously as a downstream metabolite of leucine—a branched chain amino acid (BCAA)—through its catabolic pathway. The metabolic origin of HMB was first characterized in 1988 when Nissen and colleagues investigated leucine metabolism, identifying HMB as a cleavage product derived from α-ketoisocaproic acid (α-KIC), with approximately 5% of dietary leucine undergoing conversion to HMB. Subsequent clinical trials by Nissen’s group further elucidated HMB’s mechanistic role in attenuating muscle protein breakdown, particularly under catabolic conditions such as intensive exercise training or age-related sarcopenia [6,7,8,9]. These foundational studies provided a scientific basis for the development and utilization of HMB.

Subsequent mechanistic studies have elucidated HMB’s dual role in skeletal muscle homeostasis: (1) promoting myofibrillar protein synthesis via mTORC1 signaling activation [10], and (2) suppressing proteolysis through ubiquitin-proteasome system inhibition [6,11]. These molecular effects translate to clinically measurable outcomes, including increased lean mass, optimized fat-free body composition, and attenuated age-related muscle atrophy. Notably, HMB supplementation demonstrates pleiotropic benefits, extending beyond musculoskeletal health to encompass immunomodulatory effects [9,12]. To date, HMB has been evaluated for safety by regulatory agencies (e.g., the Food and Drug Administration (FDA)’s Generally Recognized as Safe (GRAS) designation) and is now widely applied in food, medicine, and healthcare products.

Given its established safety profile and multifaceted bioactivity, HMB has emerged as a promising candidate for future applications. Numerous studies have explored its safety, metabolic pathways, synthesis, detection technologies, and physiological functions. Numerous studies have explored its safety, metabolic pathways, synthesis, detection technologies, and physiological functions. With the goal of supporting further development within the HMB industry, this review aims to sort out the research progress of the safety evaluation and efficacy mechanism of HMB and summarize the progress of production technologies such as microbial production and chemical synthesis of HMB. The development of detection technologies of HMB were also addressed.

## 2. The Safety of HMB

**Figure 1:**
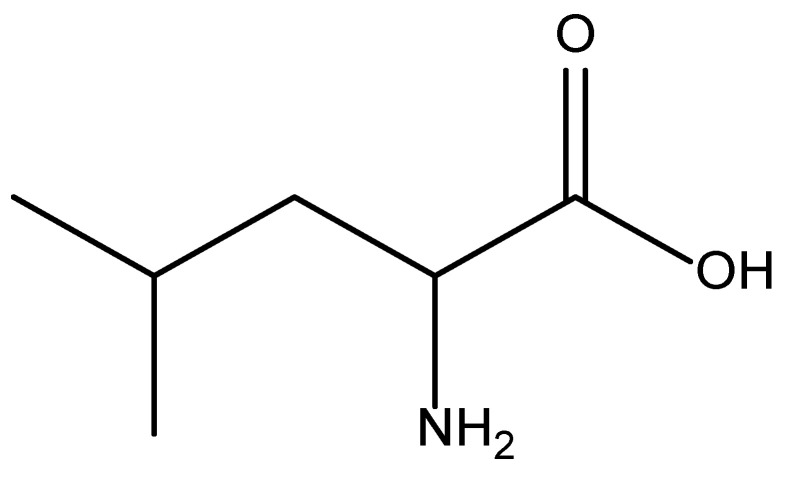
The Structural Formula of HMB.

β-Hydroxy-β-methylbutyrate (HMB, C_5_H_10_O_3_) is a five-carbon branched-chain organic acid with the structural formula shown in Figure 1. It is commercially available in industrial applications as two forms owing to its inherent chemical reactivity, namely calcium-bound HMB (CaHMB) and free acid HMB (HMB-FA) [13]. CaHMB is a salt formed by the combination of HMB and calcium ions and is the most widely used form in industry. It exists as a white crystalline powder that is easily soluble in water and is often formulated into powders or capsules to facilitate storage and use. Studies have demonstrated that CaHMB is efficiently absorbed in the body, providing a steady supply of HMB [14,15]. HMB-FA, the unbound form of HMB, can exist as either a liquid or a gel. Research indicates that this form may have a higher absorption rate than CaHMB [16]. Fluller et al. compared the effects of the free acid gel form of HMB and CaHMB on the clearance rate and availability of HMB in humans and found that the free acid gel form of HMB can increase the concentration of HMB in the plasma more rapidly and demonstrates higher efficiency in terms of retention and utilization within the body, compared with CaHMB. The experiment was carried out by comparing three forms: CaHMB (gelatin capsule, 1 g), the equivalent HMB free acid gel swallowed (FASW), and the free acid gel held sublingually for 15 s and then swallowed (FASL). The results indicated that for both FASL and FASW, the time taken for HMB to reach its peak concentration in the plasma was much shorter than that of CaHMB in capsule form (38 min and 38 min, respectively, compared to 128 min, with *p* < 0.0001). Moreover, the peak concentrations were also higher (249 μmol/L and 239 μmol/L, respectively, compared to 131 μmol/L, with *p* < 0.0001). Additionally, the half-lives were 2.50 h, 2.51 h, and 3.17 h for FASW, FASL, and CaHMB, respectively (*p* < 0.004). The free acid gel led to quicker attainment of and greater plasma concentrations (an increase of +185%) as well as improved clearance (an improvement of +25%) of HMB from the plasma. These outcomes imply that HMB free acid gel might enhance the bioavailability and efficacy of HMB in tissues. However, further verification is still necessary [17].

Since its discovery, the safety of HMB has been extensively investigated. Nissen et al. evaluated its safety by supplementing the diet of three pigs, each weighing around 20 kg with 100 mg of CaHMB for four days. The study revealed no adverse effects, as there were no alterations in blood cell counts, organ weights, or histological abnormalities [7]. In a subsequent study, Nissen et al. evaluated the safety of HMB in humans. They administered 3 g of HMB daily to a diverse group of individuals, including men and women, young and old, exercising and non-exercising subjects, for 3 to 8 weeks. Measurements of organ and tissue function, blood chemistry, hematology, emotional perception, and HMB tolerance all indicated that HMB was safe for human ingestion. It was also noted that HMB is essential for growth processes [8].

Several other studies have provided further corroboration for the safety of HMB [8,14,18,19,20]. Pitchford et al. carried out three genotoxicity assays, including the bacterial reverse mutation test, the in vitro mammalian cell chromosome aberration test, and the mammalian erythrocyte micronucleus test. Their findings demonstrated that CaHMB did not possess genotoxic properties [21]. Baxter et al. conducted a 91-day experiment in which they supplemented male and female mice with 1%, 2%, or 5% CaHMB. The results revealed that there were no adverse impacts on body weight, food consumption, clinical chemistry, or hematology [14]. Kreider et al. investigated high-intensity training participants who were supplemented with HMB at doses of 0, 3, and 6 g/day for 28 days, and ascertained that there were no significant disparities among any of the 31 blood and metabolic biochemical parameters [22]. Gallagher et al.’s study suggested that when the supplementation dose of HMB was ≤76 mg·kg^−1^·d^−1^, there were no adverse effects on hepatic enzyme function, lipid profile, renal function, or the immune system [23]. Additionally, Rathmacher et al. undertook a study, wherein a single supplementation of up to 50 g of HMB elicited no adverse effects, furnishing additional support for the safety of HMB [24]. Long-term studies have also attested to its safety. For instance, one-year studies involving older adults supplemented with 2–3 g/day exhibited no influence on liver, kidney, or lipid markers [14]. A 91-day subchronic toxicity study was carried out on Sprague Dawley rats by Fuller et al., in 2014 [25]. The experimental rats were fed daily with diets containing 0%, 0.8%, 1.6%, and 4.0% HMBFA of the diet by weight every day. Their clinical conditions, body weight changes, food consumption, hematology, clinical chemistry, urinalysis, pathological examinations, and histological examinations were evaluated. It was found that there were no deaths or abnormal clinical manifestations, no adverse changes in clinical chemistry or hematology attributable to the administration of HMBFA, and no tissue abnormalities caused by HMBFA. All measured parameters were within the normal physiological range for the animals or were in line with what was expected for the studied population. In conclusion, the no-observed-adverse-event-level (NOAEL) for HMBFA was the highest dose administered, that is, 4% of the diet, which corresponded to intakes of 2.48 g/kg BW·d and 2.83 g/kg BW·d for male and female rats, respectively. When converted to the equivalent human dosage based on body surface area, it would be 402 mg/kg BW·d for men and 459 mg/kg BW·d for women. This study suggests that HMBFA is safe when used within the recommended dosage, providing a scientific foundation for the safety of HMBFA [25].

Although HMB has been proven to be safe and non-toxic by many studies, and has many positive effects such as promoting muscle growth, there were still some studies that did not support the HMB’s efficacy; for example, Holecek speculated that HMB may have some adverse effects in other tissues. The stimulation of protein synthesis and the inhibition of proteolysis by HMB reduce the release of various amino acids from muscles into the bloodstream and may decrease the availability of these amino acids in visceral tissues. It may lead to a decrease in the concentration of glutamine in the plasma of critically ill patients [14,26]. The reasons for these contradictory results may be attributed to the differences among the subjects, the adequacy of sample collection, the disparities in testing methods, and many other factors.

As a novel food ingredient, HMB has obtained regulatory approval in many countries (Table 1). In 2005, the U.S. FDA granted HMB the GRAS (Generally Recognized as Safe) status, authorizing its application in both foods for special medical purposes and conventional foods. Subsequently, in 2009, the FDA approved an elevated use level of up to 6 g/day. In 1997, the EU approved HMB as a food ingredient. China, on the other hand, approved CaHMB as a new food resource in 2011, with a daily intake limit of ≤3 g. In 2017, China further extended its application scope to beverages, dairy products, confectionery, and baked goods. In 2022, the Chinese Health Commission proposed a new allowable daily intake of ≤6 g/day [27].

Although CaHMB has been authorized in many countries, an international consensus has yet to be reached regarding its optimal safe dosage. Current research indicates that a daily intake of 3–6 g/day is generally regarded as safe. However, further investigations are required to ascertain the optimal dosage as well as the overall efficacy of HMB [26].

## 3. The Metabolism of HMB

**Figure 2:**
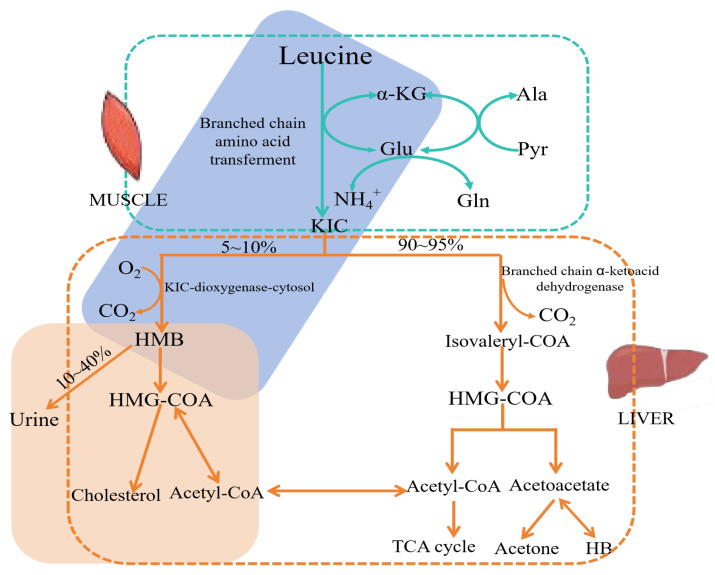
The metabolism of HMB.

As a metabolite of leucine, the metabolic pathway of HMB has been comprehensively explored in humans and animals. The biosynthesis of HMB initiates when leucine undergoes transamination to α-ketoisocaproic acid (α-KIC) via branched-chain amino acid transferase (BCAA). However, only a small proportion of leucine (around 5–10%) enters this pathway, as approximately 80% of leucine is diverted towards protein synthesis rather than α-KIC formation [28,29]. Once leucine has been metabolized to α-KIC, the latter can either be transformed into isovaleryl-CoA by α-ketoacid dehydrogenase within the mitochondria or be converted into HMB in the cytosol by α-ketoisocaproate dioxygenase. The majority of α-KIC is channeled towards isovaleryl-CoA production, which subsequently undergoes further metabolism into acetyl-CoA and acetoacetyl-CoA. Under normal circumstances, approximately 5–10% of α-KIC is converted into HMB [3,23,26]. Once formed, roughly 10–40% of HMB is excreted in the urine, while the remainder is further metabolized to HMB-CoA, which is then esterified into β-hydroxymethylglutaryl-CoA (HMG-CoA) and ultimately utilized for cholesterol synthesis. It is noteworthy that the endogenous conversion of leucine into HMB is rather limited, as healthy adults are capable of producing only 0.3–0.4 g of HMB per day (Figure 2) [28]. Consequently, supplementary HMB is often necessary to attain higher levels. Although there is evidence suggesting that HMB carbon is promptly incorporated into cholesterol, the precise proportion of cholesterol carbon originating from HMB remains elusive. The half-life of HMB differs among species: it is approximately 1 h in rats, 2 h in pigs, and 3 h in sheep, indicative of its rapid metabolism [7].

## 4. Advances in HMB Synthesis

The synthesis of HMB has been accomplished through both chemical and biological methods. Currently, chemical synthesis serves as the primary method for industrial-scale production, while biosynthetic methods are still under development.

### 4.1. Chemical Synthesis of HMB

Chemical synthesis remains the predominant method for β-hydroxy-β-methylbutyrate (HMB) production, typically involving halogenation and esterification reactions. Common raw materials include diacetone alcohol (C_6_H_12_O_2_) and sodium hypobromite (BrH_2_NaO). Zhang et al., demonstrated a halogenation method that utilized BrH_2_NaO and C_6_H_12_O_2_ to synthesize HMB, which was then purified to produce CaHMB [26]. A similar methodology reported by Tan et al., achieved 49.6% CaHMB yield through acidification (pH 2.0–2.5) and isobutanol extraction [30]. Although this approach is prevalently adopted, it suffers from certain drawbacks, such as the generation of chloroform and other deleterious by-products.

Esterification, owing to its merits of lower pollution and enhanced product stability, has emerged as a favored chemical synthesis route. Meng et al. detailed an experimental plant capable of yielding 1.5 tons of HMB per month, using C_6_H_12_O_2_ and C_4_H_8_O_2_ as raw materials [31]. Tang et al. adopted glacial acetic acid (C_2_H_4_O_2_) peroxidized with sulfuric acid (H_2_SO_4_) and hydrogen peroxide (H_2_O_2_), followed by distillation and oxidation, to synthesize 3-methyl-3-hydroxybutyric acid methyl ester, which was then hydrolyzed with alkali to procure HMB [32].

In recent years, new technologies, such as electrolytic synthesis, have emerged, simplifying the production process, reducing by-products, and enhancing economic and environmental benefits. For example, C_4_H_10_O and CO can be converted to HMB or its salts in aqueous mixtures. Additionally, combined chemical-enzymatic approaches are being explored for HMB production and may offer unique advantages in the future.

### 4.2. Biotransformation of HMB

Biotransformation methods have gained prominence in functional compound synthesis owing to their alignment with green chemistry approaches and sustainable production principles. Notably, microbial fermentation systems and whole-cell catalytic processes have demonstrated successful applications in β-hydroxy-β-methylbutyrate (HMB) biosynthesis.

All studies on the microbial fermentation of HMB to date have employed *Galactomyces reessii* strains. In 1981, Hasegawa et al. first reported the conversion of isobutyric acid to HMB using the *G. reessii* CBS 179.60 strain. In 1997, Lee et al., developed a two-step supplementation batch fermentation method with the same strain. By using glucose and β-methylbutyric acid (MBA) as substrates, maintaining a pH of 6.5 during the growth phase and 7.0 during the production phase, and setting a substrate concentration of 20 g/L, the system achieved an HMB yield of 38 g/L after 136 h [33,34]. Fei et al., optimized the fermentation conditions and, using MBA as the substrate, achieved a conversion rate of 57.3% and an HMB concentration of 29.0 g/L after 102 h [35].

Although microbial fermentation has achieved notable success in HMB production, it remains constrained by critical limitations. Specifically, the β-methylbutyric acid substrate is characterized by its elevated cost and malodorous nature, and the process itself is fraught with complexity, rendering it inappropriate for industrial-scale production.

With the rapid progression of synthetic biology, researchers are now capable of precisely designing and modifying microorganisms and enzymes via gene editing and metabolic engineering. In 2021, Gao et al., devised a whole-cell catalytic method for HMB production by heterologously expressing L-amino acid deaminase (L-AAD) from *Proteus vulgaris* and 4-hydroxyphenyl-pyruvate dioxygenase (4-HPPD) from *Rattus norvegicus* in *E. coli*. This system catalyzed the conversion of leucine to α-KIC and subsequently to HMB, attaining a conversion rate of 80% and a yield of 0.257 g/(L·h) [36]. Although this approach shows promise, it remains costly due to the use of leucine as a substrate. In 2024, Huang et al., developed a de novo synthesis of HMB by incorporating heterologous enzyme systems into *Escherichia coli*, which combined the mevalonate pathway with myxobacterial iso-fatty acid biosynthesis to convert acetyl-CoA into HMB. the engineered strain achieved an HMB production titer of 17.7 g/L using a bench-top bioreactor, with glucose as the carbon source and a glucose conversion rate of 0.16 g/g [37]. Although the engineered strain after modification has achieved de novo synthesis of HMB using glucose as the carbon source and exhibits the advantages of being able to produce HMB using other renewable carbon sources such as xylose, glycerol, and acetate, it still employs *Escherichia coli* as the host strain. *Escherichia coli*, as substrate cells, has many advantages such as low cost, easy cultivation, and rapid growth rate. However, *E. coli* may produce endotoxins like lipopolysaccharides in the cell wall, which are difficult to completely remove during separation and purification. This is unfavorable for the application of fermentation products in food. Therefore, it remains necessary to use food-safe hosts (such as those with GRAS certification) in HMB production [38]. Nevertheless, this work provides valuable insights for the future of HMB biosynthesis.

## 5. The Determination Methods of HMB

**Figure 3:**
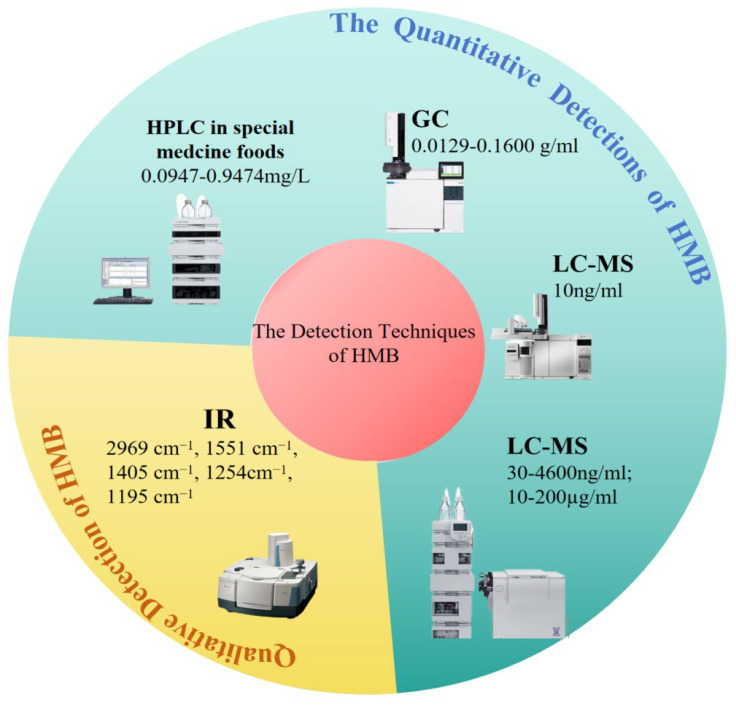
The Detection Method of HMB/CaHMB.

In recent years, with the growing application of CaHMB in specialized medical formulas and functional foods, researchers have directed their attention to establishing quality control methods for this compound. A variety of analytical techniques have been developed to quantify HMB and CaHMB, such as gas chromatography (GC), gas chromatography-tandem mass spectrometry (GC-MS), high-performance liquid chromatography (HPLC), HPLC-mass spectrometry (HPLC-MS), and atomic absorption spectrometry (AAS) (Figure 3).

### 5.1. High-Performance Liquid Chromatography (HPLC)

Among the available methods, HPLC has been the most prevalently utilized for HMB determination. For instance, Lee et al., Fei et al., and Gao et al., all employed HPLC in their studies on HMB production [33,35,36]. Yang et al. established an HPLC method for quantifying CaHMB in foods formulated for special medical purposes. The sample preparation entailed extraction with 0.1 mol/L HCl, succeeded by centrifugation and filtration. Separation was achieved on a Caprisil C18-P column (250 mm × 4.6 mm, 5 μm) using a mobile phase of 0.05 mol/L potassium phosphate buffer (pH 3.0) and acetonitrile (95:5). Detection was conducted with an Ultraviolet (UV) detector, and the method was capable of detecting CaHMB within the concentration range from 0.0947 to 0.9474 mg/L. The Limit of Detection (LOD) refers to the minimum concentration or quantity of a substance that an analytical method can reliably detect, which directly reflects the sensitivity of the method. It is a very crucial indicator of the detection method. In this study, the LOD was 0.09 μg/g [39]. Similarly, Zhu et al., developed an HPLC method possessing an LOD of 0.02 g/100 g. This method employed a Phenomenex Gemini C18 column (250 mm × 4.6 mm, 5 μm), with a mobile phase of 0.01 mol/L sodium heptanesulfonate and methanol (95:5). The flow rate was 0.8 mL/min, the column temperature was set to 30 °C, and detection was performed at 214 nm using a diode array detector. The method was simple, sensitive, and accurate, rendering it suitable for detecting CaHMB in sports nutrition products [40]. Baxter et al. similarly developed a simple, accurate, and precise HPLC quantification of HMB in liquid nutritional products. The quantitation limit was 90 mg/kg [41].

### 5.2. Gas Chromatography (GC)

Zhang et al., developed a GC method for HMB determination. The experimental conditions comprised a DB-624 column (30 m × 0.45 mm, 2.55 μm), flame ionization detection (FID), and high-purity nitrogen as the carrier gas with a flow rate of 1.0 mL/min. The column temperature was initially maintained at 150 °C and then increased at a rate of 10 °C/min until it reached 240 °C, where it was held for 1 min [42].

### 5.3. HPLC-MS/MS and GC-MS/MS

Deshpande et al., devised a sensitive and specific HPLC–MS/MS method for the determination of HMB in small volumes of rat plasma. The limit of detection (LOD) was 30 ng/mL, and the detection range extended from 30 to 4600 ng/mL (R^2^ ≥ 0.995) [43]. Huang et al. established an LC–MS/MS method for detecting HMB in dairy products, with an LOD of 50.0 μg/kg and a detection range of 10.0–200.0 μg/L. This method successfully mitigated matrix interference from milk powder, augmenting the sensitivity of HMB detection in dairy products [42]. Similarly, Wang et al. devised a GC-MS/MS method for HMB determination, attaining an LOD of 10 ng/mL [44].

### 5.4. Infrared Absorption Spectroscopy

In addition to chromatographic methods, Yang et al., established a qualitative determination of CaHMB by means of infrared absorption spectroscopy. They ascertained characteristic absorption peaks at 2969 cm^−1^, 1551 cm^−1^, 1405 cm^−1^, 1254 cm^−1^, and 1195 cm^−1^, thus demonstrating the potential of this technique for CaHMB identification [39].

## 6. The Physiological Functions and Applications of HMB

As a metabolite of leucine, HMB has been demonstrated to exhibit diverse physiological benefits, such as anticatabolic activity, anabolic stimulation, and lipolytic regulation [45]. Supplementation with HMB enhances muscle protein synthesis, reduces protein catabolism, improves muscle mass, mitigates muscle damage, improves lean body mass, diminishes fat deposition, bolsters strength and endurance, alleviates inflammatory responses, and stabilizes cell membranes [46]. Moreover, HMB augments animal production performance, enhances intestinal health, and potentiates immunity, rendering it widely utilized in specialized medical foods, sports nutrition, and animal feed (Table 2).

### 6.1. Muscle Preservation and Disease Treatment

HMB is efficacious in preventing muscle loss attributable to aging, cancer cachexia, AIDS, and endotoxemia, endowing it with significant value in the treatment of diseases associated with weight loss, especially sarcopenia [3,28]. Studies have demonstrated that combining HMB with amino acids, such as glutamine and arginine, assists in maintaining lean body mass and reversing cancer-induced weight loss in patients with chronic diseases [47]. For instance, Clark et al., noted a significant augmentation in lean body mass and an amelioration in the immune status of subjects following an 8-week supplementation of an arginine (Arg) and glutamine (Gln) and HMB mixture (14 g Arg + 14 g Gln + 3 g HMB/d) in AIDS patients [48]. The same mixture (14 g Arg + 14 g Gln + 3 g HMB/d) was studied to ascertain whether it could reverse the cachexia process in patients with stage IV cancer. The result revealed that the mixture was efficacious in increasing FFM [49]. Similarly, Sipahi et al. observed that HMB, in combination with Gln and Arg, promoted wound healing in diabetic dialysis patients [50]. Cramer et al. found that 12 weeks of CaHMB supplementation in patients with sarcopenia led to significant improvements compared to controls [51].

Yang et al., conducted a study involving elderly patients with colon cancer, demonstrating that HMB supplementation remarkably improved the nutritional status and post-surgical recovery, as evidenced by elevated hemoglobin and albumin levels 30 days after the operation [52]. HMB’s affirmative influence on protein synthesis and its diminution of protein degradation underpin its capacity to bolster recovery and muscle preservation during illness.

### 6.2. Impact on Body Composition and Physical Performance

In older adults, HMB supplementation has been demonstrated to positively modulate body composition, muscle mass, and strength [53]. Nicolaas et al. concluded that HMB is beneficial for arresting muscle loss due to prolonged bed rest in older adults [54]. A study involving community-dwelling older women indicated that daily oral supplementation with 1.5 g of CaHMB over eight weeks significantly enhanced muscle strength and physical function [15]. In a year-long study, supplementation with HMB, L-arginine, and L-lysine augmented protein turnover and lean tissue in elderly participants [55]. Zou et al., ascertained that while HMB alone might not impact body composition, its combination with resistance exercise improved lower extremity muscle strength in older adults [56].

### 6.3. Ergogenic Use in Young Trained/Untrained Subjects

HMB is widely employed as an ergogenic aid by bodybuilders and strength athletes. Research corroborates its efficacy in enhancing exercise performance and fostering skeletal muscle hypertrophy [57,58]. Nissen et al. found that supplementation of 1.5 to 3 g/day of HMB, in conjunction with weightlifting, mitigated exercise-induced proteolysis and muscle damage, leading to greater muscle function gains [6]. Panton et al., noted that supplementation of 3.0 g/day of HMB during resistance training led to more pronounced increases in upper body strength, fat-free weight, and a reduction in fat percentage [59]. Additionally, Jowko et al., demonstrated that daily supplementation with 3 g of HMB combined with creatine enhanced muscle hypertrophy and diminished markers of muscle damage [45]. Wilson et al., ascertained that HMB-free acid could reduce markers of exercise-induced muscle damage and improve recovery in resistance-trained men [16]. and Sadi et al.’s research also showed that the supplementation of HMB-FA can enhance the effects of resistance training by increasing anabolic hormones [60].

However, some studies have documented no significant effects of HMB on athletic performance [22,61,62,63,64,65]. Kreider et al., found that 3 g/day of CaHMB supplementation during training in NCAA football players had no significant impact on strength or body composition [22]. Slater et al., evaluated the effects of oral HMB supplementation on the training responses of resistance-trained male athletes. The athletes were randomly and double-blindly assigned to receive HMB in standard encapsulation (SH), HMB in time-release capsules (TRH), or a placebo (P). Subjects ingested 3 g·day^−1^ of HMB or the placebo for 6 weeks. The test was used to assess body mass, body composition, 3-repetition maximum strength, and biochemical parameters, including markers of muscle damage and muscle protein turnover. The data indicate that 6 weeks of HMB supplementation, whether in SH or TRH form, does not affect the changes in strength and body composition of strength-trained athletes in response to resistance training [64]. Shirato et al. found that ingestion of combined HMB and whey protein does not have an effect to inhibit muscle strength loss and soreness, and decrease in muscle damage markers after eccentric exercise in comparison with HMB and whey protein alone [62]. These mixed results indicate that HMB’s effectiveness may differentiate depending on the individual and specific training conditions.

### 6.4. Applications in Animal Nutrition

As a promising nutritional additive for livestock and poultry, HMB has demonstrated significant effects in improving animal production performance and carcass traits, promoting muscle growth, enhancing immune function and intestinal health, as well as reducing mortality [66,67]. Its mechanisms of action may involve the regulation of protein metabolism, lipid metabolism, mitochondrial biogenesis, and cholesterol synthesis [5,7,68]. For instance, HMB can upregulate the expression of insulin-like growth factor 1 (IGF-1) and growth hormone to enhance protein conversion efficiency [69]. It can promote myoblastogenesis through the mitogen-activated protein kinase/extracellular signal-regulated kinase (MAPK/ERK) and phosphoinositide 3-kinase (PI3K)/Akt pathways facilitated by IGF-I [70]. Additionally, HMB can reduce protein degradation by attenuating the activation and increased gene expression of the ubiquitin-proteasome proteolytic pathway induced by the proteolysis-inducing factor (PIF) [71].

As a nutritional additive for livestock and poultry, HMB has been proven to be effective in a variety of animals. In piglets, HMB supplementation exerts a positive influence on newborn weight, colostrum quality, and growth performance from weaning to fattening. Tatara et al., demonstrated that feeding sows 10 g/day of CaHMB before farrowing increased piglet birth weight by 22% and reduced the time to market for fattening pigs [72]. Wan et al., found that HMB supplementation enhanced the expression of MyHC-IIb mRNA and fast MyHC protein, improving muscle quality in piglets [73].

In addition to piglets, HMB demonstrated potential for utilization in sheep feed. Zabek et al. found that although HMB supplementation did not influence total weight gain in goats, it enhanced feed efficiency and weight gain per unit of feed [74]. In laying hens, Tomaszewska et al., documented that HMB supplementation led to positive changes in bone microarchitecture and muscle quality, though not all effects were uniformly beneficial [75].

A review by Nissen et al. found that HMB inhibited muscle proteolysis by 80% in animal models, while protein synthesis increased by 20% [7]. These findings underscore HMB’s value in enhancing animal growth, muscle development, and immune function.

**Table 2:** Research on HMB as a Nutritional Supplementation.

Study Design	Results	Author (Year)
68 HIV infected patients, HMB 3 g/d + 14 g/d Arg + 14 g/d Gln, 8 weeks	Increased CD_3_ and CD_8_ cells, decreased HIV viral, gained BW and LBM, and altered the course of lean tissue loss in patients with AIDS associated wasting	Clark et al., 2000 [39]
32 patients with solid tumors, 3 g/d HMB + 14 g/d Arg + 14 g/d Gln, 24 weeks	Increased the FFM of advanced (stage IV) cancer	May et al., 2002 [49]
11 diabetic dialysis patients, 2.6 g/d HMB + 14.8 g/d Gln + 14.8 g/d Arg, 4 weeks	Had a positive contribution to the wound healing	Sipahi et al., 2013 [50]
Malnourished and sarcopenic men and women, 3.0 g/d HMB + 40 g/d protein + 998 IU vitamin D_3_, 4 weeks	Improved leg muscle strength and quality in not severe sarcopenia	Cramer et al., 2016 [51]
patients with I–IIIstage colon cancer, 3 g/d HMB + nutritional supplement, 5 weeks	Improved PG-SGA and MNA scores, greater hemoglobin and albumin	Yang et al., 2022 [52]
24 healthy older adults (60–79 years old) during 10 days of bed rest, 3 g/d, 8 weeks	Prevented the decline in LBM	Deutz et al., 2013 [54]
39 women and 38 men, 2 g/d HMB + 5 g/d Arg + 1.5 g/d Lys, 1 year	Increased lean tissue; increased body cell mass and lean mass; increased protein turnover	Baier et al., 2008 [55]
healthy older women, 1.5 g/d HMB, 8 weeks	Improved some muscle strength and physical functioning parameters	Tian et al., 2021 [15]
20–40 years old women and men, 3.0 g/d + 3 times per week RT, 4 weeks	Increased body strength, fat-free weight; decreased fat percentage	Panton et al., 2000 [59]
19–29 years men, 0, 1.5 or 3.0 g/d + normal, 117 g/d and 175 g/d Protein, 7 weeks	Either 1.5 or 3 g HMB/day could partly prevent exercise-induced proteolysis and/or muscle damage and improved in muscle function associated with resistance training	Nissen et al., 1996 [6]
37 untrained college-aged men, 0, 3 or 6 g HMB + 3 d·wk^−1^ RT, 8 weeks	Increased peak isometric, isokinetic torque values and FFM; decreased plasma CPK activity	Gallagher et al., 2000 [23]
20 RT males, 3 g/d HMB-FA	Blunt increases in muscle damage and prevent declines in perceived readiness	Wilson et al., 2013 [16]
16 healthy men, 3 g/d HMB + RT, 6 weeks	Increased 1RM leg press; decreased CORT and ACTH; improved GH and IGF-1	Asadi et al., 2017 [60]
20 sows, 4 g/d HMB, from the 35th day of gestation to parturition	Increased birth weight; decreased the rate of stillborn piglets and the feed intake	Wan et al., 2016 [73]
24 Alpine goats, 50 mg/kg HMB, 60 days	Higher body weight, more favorable musculature development	Zabek et al., 2016 [74]
48 Bovans Brown, 0.02% HMB in diet	Increased mean daily, total egg weight, trabecular bone structure; decreased cholesterol content and alterations in the fatty acid profile	Tomaszewska et al., 2024 [75]

AIDS: Acquired immunodeficiency syndrome, Arg: arginine; Gln: Glutamine; BW: Body weights; LBM: Lean body mass; FFM: Fat-free mass; Lys: Lysine; RT: Resistance training; CPK: Plasma creatine phosphokinase; 1RM: One repetition maximum; GH: Growth hormone; IGF-1: Growth factor 1; CORT: Cortisol; ACTH: Adrenocorticotropic hormone.

## 7. Conclusions and Perspective

HMB is widely acknowledged for its benefits in facilitating muscle growth and has been approved for use in general foods, specialized medical foods, and health products in many countries owing to its proven safety. Numerous studies have investigated its mechanisms of action, safety profile, and applications across different fields. Looking forward, future research should prioritize advancing sustainable and environmentally friendly production methods for HMB, particularly through bio-manufacturing processes. By using biosynthesis technology to synthesize HMB efficiently, it will lay the foundation for large-scale production of HMB in the future. Metabolic engineering technology can be utilized to optimize the metabolic pathways of microorganisms, increasing the yield of HMB. Meanwhile, continuous improvements can be made to its fermentation technology to enhance production efficiency and reduce production costs

In addition, the application scope of HMB could be further expanded across a wider array of industries, especially within the food sector. For example, HMB has shown potential in the prevention and treatment of sarcopenia and cachexia. In the future, special nutritional supplements for the elderly can be developed, as well as nutritional support products for patients with wasting diseases such as cancer and AIDS. Further exploration can be focus on combining HMB with other nutrients (such as arginine and glutamine) to enhance the effect. In the development of functional foods, the target populations can be further segmented, such as athletes, women, children and adolescents. Additionally, long-term research on the use of HMB should be further strengthened. Long-term studies will help us understand whether there may be potential health risks during the long-term use of HMB, ensuring its safety. They will also help us understand the long-term efficacy of HMB, comprehensively understand the mechanism of action, the safety and effectiveness of HMB, and guide the rational use of HMB in various fields. Furthermore, the mechanism of action of HMB’s efficacy should be further elucidated, and it is crucial to provide a more solid scientific basis for its industrial applications.

Moreover, as a nutritional supplement, the form of HMB (such as CaHMB or HMB-FA) may affect its bioavailability and efficacy. Establishing testing standards can clearly distinguish the content and purity of different forms of HMB, effectively control the quality of HMB supplements in the market, and also contribute to the accuracy of data and the comparability between them in HMB-related studies. Therefore, it is crucial to establish comprehensive standards for HMB, encompassing recommended safety doses and testing methodologies. Furthermore, it is also necessary to further refine and clarify the appropriate range of HMB addition and purity requirements across various application fields (such as food, health products, and feed) to ensure its safety and effectiveness. Developing such standards will provide a solid framework for its broader application and foster greater confidence among both consumers and industries.

## Figures and Tables

**Table 1:** Approval status of CaHMB in relevant countries/regions of the world.

Countries	Year of Approval	Forms of HMB’s Utilization	Range of Application and Dosage
China	2011, 2017, 2022	CaHMB	As a new resource food, used in foods for sports nutrition and foods for special medical purposes, ≤3 g/d (2011). As a new food raw material, used in beverages, milk and dairy products, cocoa products, chocolate and chocolate products, confectionery, and baked goods, ≤6 g/d (2017), ≤6 g/d (2022)
USA	2005, 2009	CaHMB	As medical and general foodstuffs, 3 g/d (2005), ≤6 g/d (2009)
EU	1997	CaHMB	As general food ingredients used in special dietary foods such as whole nutrition food, and nutritional supplements. ≤1.5 g/serving and ≤6 g/d
Canada	2014	CaHMB	As a new resource food and as an ingredient in natural health products, ≤1.5 g/serving and ≤6 g/d
Japan	2009	HMB	As functional food, 1.6~4.8 g/d

1 g CaHMB contains approximately 0.8 g HMB.

## Data Availability

No new data were created or analyzed in this study. Data sharing is not applicable to this article.
